# Baseline chest X-ray in coronavirus disease 19 (COVID-19) patients: association with clinical and laboratory data

**DOI:** 10.1007/s11547-020-01272-1

**Published:** 2020-09-07

**Authors:** Marco Gatti, Marco Calandri, Matteo Barba, Andrea Biondo, Carlotta Geninatti, Stephanie Gentile, Marta Greco, Vittorio Morrone, Clara Piatti, Ambra Santonocito, Sara Varello, Laura Bergamasco, Rossana Cavallo, Rosario Di Stefano, Franco Riccardini, Adriana Boccuzzi, Giorgio Limerutti, Andrea Veltri, Paolo Fonio, Riccardo Faletti

**Affiliations:** 1 Radiology Unit, Department of Surgical Sciences, University of Turin, Via Genova 3, 10126 Turin, Italy; 2 Radiology Unit, Department of Oncology, San Luigi Gonzaga Hospital, University of Turin, Orbassano, TO Italy; 3 Department of Surgical Sciences, University of Turin, Turin, Italy; 4 Laboratory of Microbiology and Virology, Department of Public Health and Pediatrics, Città della Salute e della Scienza Hospital, University of Turin, Turin, Italy; 5 Department of Oncology, San Luigi Gonzaga Hospital, University of Turin, Orbassano, TO Italy; 6 Emergency Department, San Luigi Gonzaga University Hospital, Orbassano, TO Italy; 7 Department of Medical Science, University of Turin, Turin, Italy; 8 Department of Radiology, S.C. Radiodiagnostica Ospedaliera, Turin, Italy

**Keywords:** Chest X-ray, Coronavirus disease 2019 (COVID-19), Severe acute respiratory syndrome coronavirus 2 (SARS-CoV-2), Real-time reverse transcriptase-polymerase reaction chain test (RT-PCR), Laboratory test, Sensitivity

## Abstract

**Purpose:**

To assess the reliability of CXR and to describe CXR findings and clinical and laboratory characteristics associated with positive and negative CXR.

**Methods:**

Retrospective two-center study on consecutive patients admitted to the emergency department of two north-western Italian hospitals in March 2020 with clinical suspicion of COVID-19 confirmed by RT-PCR and who underwent CXR within 24 h of the swab execution. 260 patients (61% male, 62.8 ± 15.8 year) were enrolled. CXRs were rated as positive (CXR+) or negative (CXR−), and features reported included presence and distribution of airspace opacities, pleural effusion and reduction in lung volumes. Clinical and laboratory data were collected. Statistical analysis was performed with nonparametric tests, binary logistic regression (BLR) and ROC curve analysis.

**Results:**

Sensitivity of CXR was 61.1% (95%CI 55–67%) with a typical presence of bilateral (62.3%) airspace opacification, more often with a lower zone (88.7%) and peripheral (43.4%) distribution. At univariate analysis, several factors were found to differ significantly between CXR+ and CXR−. The BLR confirmed as significant predictors only lactate dehydrogenase (LDH), C-reactive protein (CRP) and interval between the onset of symptoms and the execution of CXR. The ROC curve procedure determined that CRX+ was associated with LDH > 500 UI/L (AUC = 0.878), CRP > 30 mg/L (AUC = 0.830) and interval between the onset of symptoms and the execution of CXR > 4 days (AUC = 0.75). The presence of two out of three of the above-mentioned predictors resulted in CXR+ in 92.5% of cases, whereas their absence in 7.4%.

**Conclusion:**

CXR has a low sensitivity. LDH, CRP and interval between the onset of symptoms and the execution of CXR are major predictors for a positive CXR.

## Introduction

Coronavirus disease 2019 (COVID-19) is caused by severe acute respiratory syndrome coronavirus 2 (SARS-CoV-2). Medical symptoms vary from asymptomatic inflammation to a wide variety of systemic and/or respiratory manifestations [1]. Laboratory findings among hospitalized COVID-19 patients include lymphopenia, high levels of aminotransaminase and elevated inflammatory markers [2]. The SARS-CoV-2 diagnostic test is the real-time reverse transcriptase-polymerase reaction chain test (RT-PCR), which is very specific but has low sensitivity (54–73%) [3].

Regarding imaging, computed tomography (CT) presents a sensitivity up to 95%, vastly outperforming RT-PCR [4]. However, the Multinational Consensus Statement from the Fleischner Society stated that CT scan should not be used for screening or as a first-line test for the diagnosis of COVID-19 [5], also because the use of a non-dedicated scanner CT requires time-consuming and laborious decontamination procedures to limit the risk of cross infection [6]. In this light, chest X-rays (CXR) can be considered as an alternative to CT, also for the easy and fast cleaning of the equipment and the large availability of portable units. In Northern Italy, the epicenter of the Italian SARS-CoV-2 pandemic, some emergency departments use CXR as the first line of triage in patients with suspected COVID-19 also because of the relatively long waiting time for RT-PCR. Although, the common opinion is that CXR may not be sufficiently sensitive for the detection of COVID-19 lung disease especially in the early stages of the pathology, there are only a few studies in the literature assessing its sensitivity in respect to the current diagnostic gold standard, RT-PCR [7–12]. Furthermore, no pertinent information is available concerning the association of CXR findings with clinical and laboratory data.

We considered the issue worth of further exploration and planned a study aimed to assess the reliability of CXR compared to RT-PCR in symptomatic patients with positive COVID-19 confirmed by RT-PCR. The secondary aim was to describe CXR findings in the context of demographics characteristics, comorbidities, and clinical/laboratory characteristics associated with positive and negative CXR.

## Materials and methods

### Study design and population

The study was piloted in agreement with the 1964 Helsinki declaration and its later amendments, was approved by the institutional review board. The requirement for informed patient consent was waived.

**Fig. 1:**
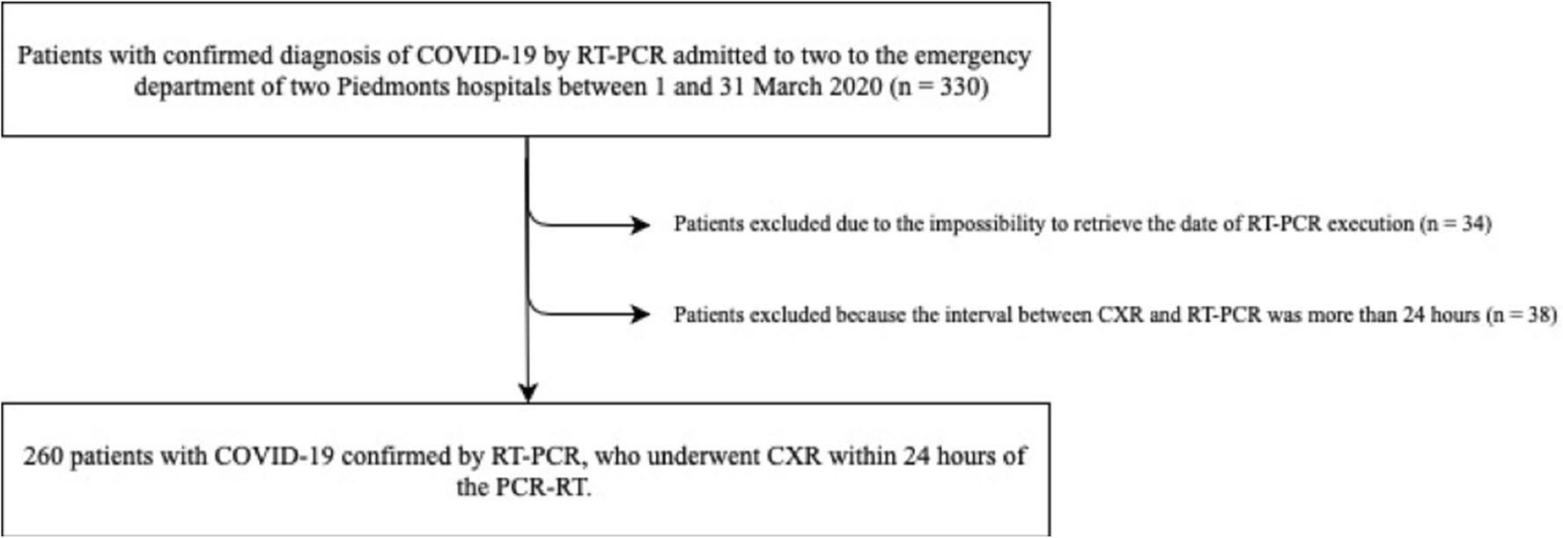
Study flow chart

This was a retrospective two center study on consecutive patients who were admitted to the emergency department of two Northern Italy hospitals between 1 and March 31, 2020 with clinical suspicion of COVID-19 confirmed by RT-PCR, and who underwent CXR within 24 h of the swab execution (Fig. 1).

### Clinical and laboratory data

The demographic data, comorbidities, clinical and laboratory data of the patients were collected in accordance with the structured report released by the “Società Italiana di Radiologia Medica e Interventistica (SIRM)” [13]. As for comorbidities, variables included in the analysis were: presence of cardiac disease, diabetes, obesity, hypertension, smoke history, ACEi/Sartan or FANS therapy. As for clinical data: Fever, Cough, Rhinitis, Dyspnea, Pharyngodynia, Myalgias, Asthenia, Conjunctivitis, Headache, Nausea, Vomit and Diarrhea.

Regarding Laboratory data: white blood cells (WBC) count, C-reactive protein (CRP), Lactate dehydrogenase (LDH), hepatic enzymes, creatin kinase (CK), blood’s pH and arterial partial pressure of carbon dioxide (PaCO2). RT-PCR protocol: extraction with QIAsymphony^®^ DSP Virus/Pathogen Midi Kit, amplification with Seegene AllplexTM 2019-nCoV Assay (target genes E, N, RdRP).

### Image acquisition and analysis

CXRs were acquired as computed or digital radiographs according to the local protocols. All CXR studies were analyzed by two observers (MC and MG with more than 5 years’ experience) using a picture archiving and communication system (PACS) workstation [Carestream Vue PACS v11.3.4 (Carestream Health, Inc, Rochester, NY)]. In the few cases of disagreement, the decision was reached by consultation with a senior radiologist (RF with more than 10 years’ experience). CXRs were rated as positive/negative, and the features reported included presence and distribution of airspace opacities, pleural effusion and reduction in lung volumes [7].

### Statistical analysis

Continuous variables were tested for normality with the Shapiro-Wilks W test. When normal, they were expressed as mean and standard deviation; otherwise, as median with first (Q1) and third (Q3) quartile. Categorical variables were presented as absolute numbers and percentages.

The univariate analysis used nonparametric tests: for 2 independent continuous variables the Mann–Whitney test, for categorical variables the *χ*^2^ test (with Yates’s correction for 2 × 2 tables) or Fisher’s exact test. Binary logistic regression (BLR) was run on variables determined as significant by the univariate analysis.

Continuous variables with significant differences were dichotomized by the ROC curve procedure to derive the regions with the strongest association with CXR+. The thresholds were obtained through three methods: maximization of the harmonic mean of Sensitivity and Specificity and of Youden’s index and minimization of the distance from the upper left corner. Agreement between the three indications was required.

Significant association with CXR+ corresponded to *p* < 0.05 and 95% CI of ORs totally above 1. The analysis was run on StatPlus: Mac v.7 (AnalysisSoft.Walnut.CA.USA).

## Results

The baseline characteristics of the study population are listed in Table 1.

**Table 1:** Baseline characteristics of the study population (*N* = 260)

*Demographic characteristics*	
Age (years)	62.8 ± 15.8
Males	61.0%
Time between onset of symptoms—CXR (days)	5 (3–8)
*Comorbidities*	
Cardiac disease	21.0%
Hypertension	46.0%
Diabetes	12.0%
Obesity	9.0%
Smoke history	32.0%
Oncologic history	15.0%
FANS	3.0%
ACEi	11.0%
Sartans	19.0%
*Clinical data*	
Fever	91.0%
Cough	66.0%
Rhinitis	3.0%
Dyspnea	37.0%
Pharyngodynia	9.0%
Myalgias	11.0%
Asthenia	13.0%
Conjunctivitis	1.0%
Headache	5.0%
Nausea	4.0%
Vomit	3.0%
Diarrhea	15.0%
*Laboratory data*	
Lymphopenia	41.0%
WBC count (10^9^/L)	6.2 (4.9–8.5)
Lymphocytes (%)	19.3%
Lymphocytes (number)	1.1 (0.8–1.5)
CRP value (mg/L)	37.7 (8.3–106.9)
LDH value (UI/L)	517 (390–669)
Alteration hepatic values	18.0%
CK elevation	19.0%
pH	7.46 (7.43–7.49)
PaCO2 (mmHg)	34 (30–37)

Using RT-PCR as gold standard, the sensitivity of CXR was 61.1% (95%CI 55–67%). The airspace opacities found at CRX were most commonly distributed in peripheral (69/159 = 43.4%) and lower zone (141/159, 88.7%) and most of the patients had bilateral involvement (99/159, 62.3%). Pleural effusion was found in 17 cases (10.7%) and lung volume reduction in 12 cases (7.5%). The CXR findings are summarized in Table 2.

**Table 2:** CXR findings

Findings of 159 CXR+	Number	%
*Distribution at CXR*		
Peripheral predominant	69	43.4
Perihilar predominant	34	21.4
Neither peripheral nor perihilar	56	35.2
Bilateral lungs	99	62.3
Upper zone involvement	37	23.3
Middle zone involvement	110	69.2
Lower zone involvement	141	88.7
No zonal predominance	32	20.1
*Other features at CXR*		
Pleural effusion	17	10.7
Reduction in lung volumes	12	7

The study sample of 260 patients with COVID-19 confirmed by RT-PCR were subdivided in two groups: 159 patients with positive CXR (CXR+) and 101 patients negative CXR (CXR−). Patients in the CXR+ group were older than in the CXR− one (66.5 ± 14.0 vs. 56.7 ± 15.5 years; *p* < 0.001), while there was an overlapping gender distribution (64.2% vs. 56.4% of male; *p* = 0.27).

The results of the univariate analysis on comorbidity, clinical and laboratory data of the two groups are listed and reported in Table 3. Several factors were found to differ significantly between CXR+ and CXR−. Regarding comorbidities, the presence of hypertension (*p* = 0.0002) and a concurrent treatment with sartans (*p* = 0.03) were more frequent in CXR+. Among clinical data, dyspnea (*p* = 0.01), myalgia (*p* = 0.004) and a longer interval between the onset of symptoms and the execution of CXR (*p* = 0.0002) were typical of CXR+. With regard to laboratory data, the presence of lymphopenia (*p* = 0.005), high level of CRP (*p* < 0.0001), LDH (*p* < 0.0001), hepatic enzymes (*p* = 0.0009) or CK (*p* = 0.02), PaCO_2_ (*p* = 0.02) and a reduction in blood’s pH (*p* = 0.04) were more common in CRX+.

**Table 3:** Comorbidity, clinical and laboratory data of CXR+ versus CXR−

Comorbidity	CXR+ (*n* = 159)	CXR− (*n* = 101)	*p* value
Cardiac disease	23.8%	16.1%	0.19
Hypertension	54.7%	29.8%	*0.0002*
Diabetes	13.7%	8.3%	0.28
Obesity	12.1%	5.4%	0.11
Smoke history	7.8%	10.8%	0.47
Oncologic history	17.8%	11.7%	0.27
FANS	3.9%	2.5%	0.71
ACEi	11.9%	8.5%	0.5
Sartans	24.1%	10.6%	*0.03*
*Clinical data*			
Onset of symptoms—CXR (days)	7 (4–8)	4 (1–7)	*0.0002*
Fever	92.3%	88.0%	0.35
Cough	61.3%	73.3%	0.06
Rhinitis	2.6%	3.0%	0.99
Dyspnea	42.9%	27.0%	*0.01*
Pharyngodynia	65%	14.0%	*0.05*
Myalgias	6.5%	18.0%	*0.004*
Asthenia	12.3%	14.0%	0.71
Conjunctivitis	0%	3.0%	0.06
Headache	3.2%	9.0%	0.05
Nausea	4.5%	4.0%	0.99
Vomit	2.6%	4.0%	0.72
Diarrhea	14.2%	16.0%	0.72
*Laboratory data*			
Lymphopenia	48.3%	27.1%	*0.005*
WBC count (10^9^/L)	6.11 (4.87–8.49)	6.2 (4.9–8.5)	0.99
Lymphocytosis	1.7%	3.0%	0.62
Lymphocytes (%)	15.3%	22.5%	*0.002*
Lymphocytes (number)	0.98 (0.74–1.29)	1.30 (0.88–1.85)	*0.0002*
CRP Elevation	93.70%	56.6%	< *0.0001*
CRP (mg/L)	77.0 (27.8–128.8)	8.1 (2.4–23.6)	< *0.0001*
LDH elevation	83.2%	28.6%	< *0.0001*
LDH value (UI/L)	599 (511–839)	277 (319–461)	< *0.0001*
Alteration hepatic values	25.4%	6.6%	*0.0009*
CK elevation	24.6%	10%	*0.02*
pH	7.47 (7.44–7.50)	7.4 5 (7.43–7.47)	*0.04*
PaCO2 (mmHg)	32.5 (30–36)	36 (33–3)	*0.02*

Significant differences were reported in italics

**Fig. 2:**
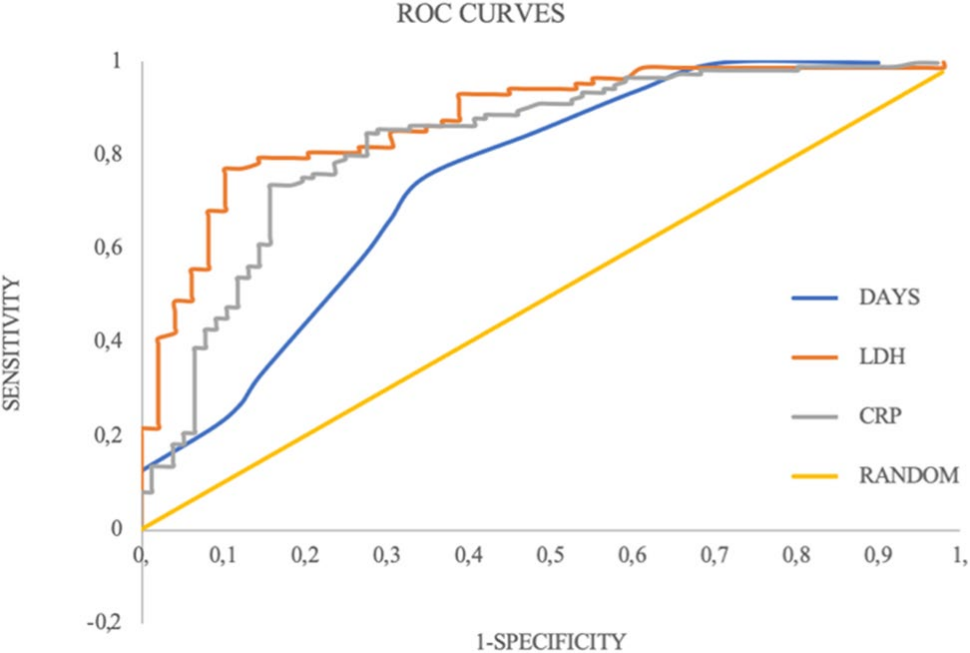
ROC curves for the discriminating ability of lactate dehydrogenase (LDH) AUC = 0.88; C-reactive protein (CRP), AUC = 0.83; and Number of days (days) between the onset of symptoms and the execution of CXR, AUC = 0.75

The significant differences exhibited by the continuous variables LDH, CRP and interval between the onset of symptoms and the execution of CXR suggested a potential predictive role for them. The ROC curve procedure (Table 4 and Fig. 2) identified as the threshold for CRX+ the range LDH > 500 UI/L, CRP > 30 mg/L and the interval between the onset of symptoms and the execution of CXR, days > 4. The BLR applied to the dichotomized variables confirmed their significant value as prognostic role (Table 5).

**Table 4:** ROC curves procedure for the discriminating ability of LDH, CRP and the interval between the onset of symptoms and the execution of CXR

Variable	AUC	Youden’s index	Threshold	SNS	SPC	PPV	NPV	DA
LDH	0.88	0.67	> 500 UI/L	0.77	0.90	0.93	0.69	0.81
CRP	0.83	0.57	> 30 mg/L	0.74	0.84	0.89	0.66	0.77
Interval symptoms—execution CXR	0.75	0.41	> 4 days	0.76	0.65	0.77	0.64	0.72

*AUC* area under the curve, *SNS* sensitivity, *SPC* specificity, *PPV* positive predictive value, *NVP* negative predictive value, *DA* diagnostic accuracy

**Table 5:** BLR applied to the dichotomized variables

Variable	*p* value	Odds ratio	LCL	UCL
LDH > 500 UI/L	0.0001	11.0	3.2	36.0
CRP > 30 mg/L	0.0006	8.0	2.5	27.0
Interval symptoms—execution CXR > 4	< 0.0001	4.0	1.3	12.5

*LCL* lower control limit; *UCL* upper control limit

**Fig. 3:**
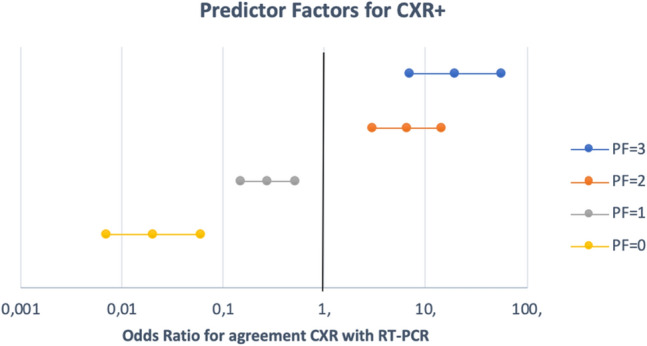
Odds Ratio 95%CIs of the three predictors for agreement of CXR with RT-PCR. 95%CIs completely above 1 correspond to positive effect on CXR+; completely below 1 correspond to adverse effects; including 1 to randomity

**Fig. 4:**
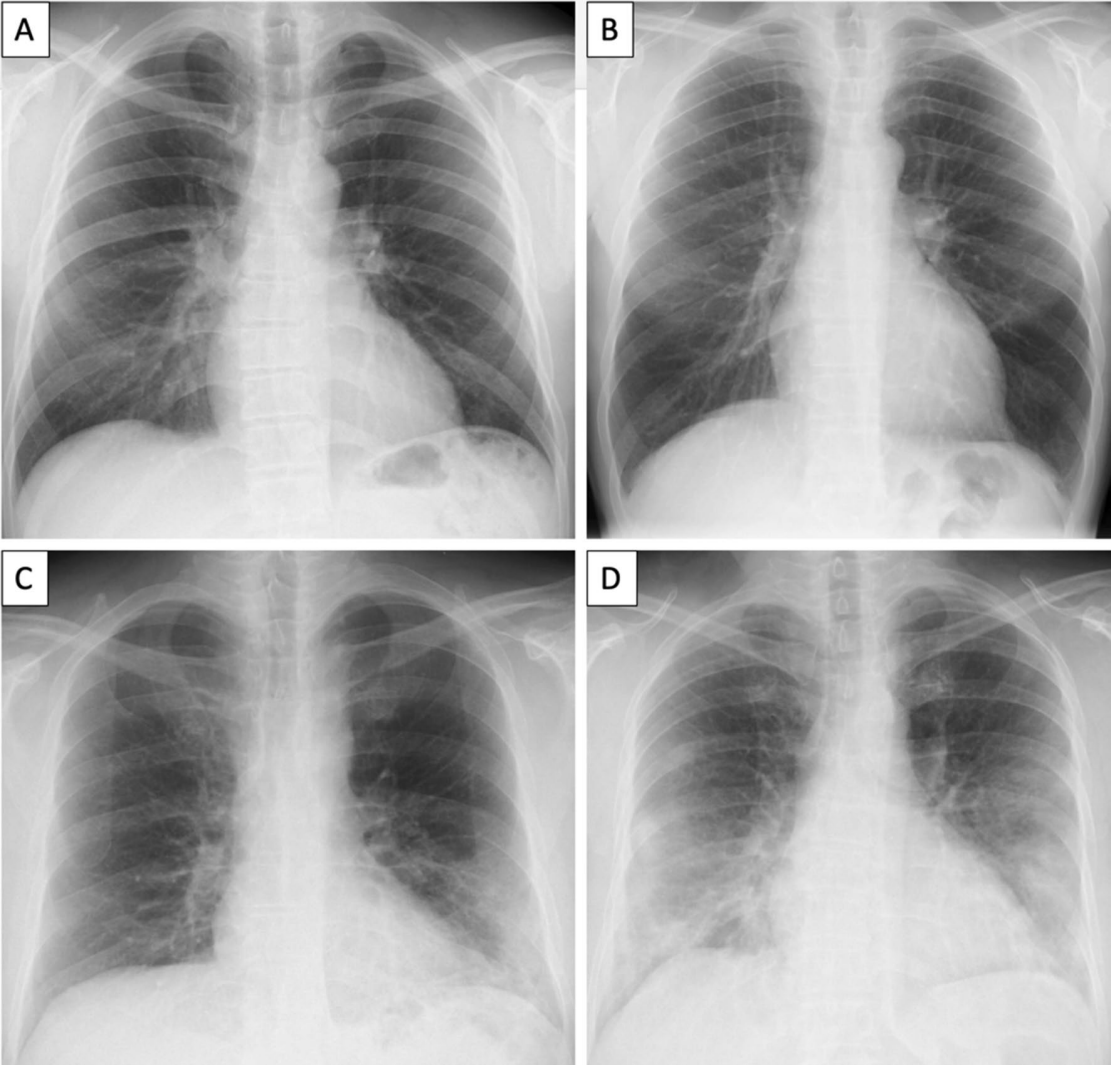
Baseline chest X-ray finding in COVID-19 patients associated with clinical and laboratory data. **a** 47-year-old man, without comorbidities, presented with fever, cough, rhinitis and conjunctivitis for 3 days. Laboratory test: Lymphocytes 1.31 (10^9^/L), CRP = 8.3 mg/L and LDH = 330 UI/L. The chest X-ray resulted negative. **b** 46-year-old man, without comorbidities, presented with fever, cough, and Pharyngodynia for 1 days. Laboratory test: Lymphocytes 0.8 (10^9^/L), CRP = 3 mg/L and LDH = 346 UI/L. The chest X-ray resulted negative. **c** 58-year-old man, without comorbidities, presented with fever and cough for 7 days. Laboratory test: Lymphocytes 0.67 (10^9^/L), CRP = 141.9 mg/L and LDH = 711 UI/L. The chest X-ray resulted positive with peripheral airspace opacification in the left lower lobe. D: 55-year-old woman, without comorbidities, presented with fever and cough for 7 days. Laboratory test: Lymphocytes 1.13 (10^9^/L), CRP = 60.9 mg/L and LDH = 784 UI/L. The chest X-ray resulted positive with neither peripheral nor perihilar airspace opacification with middle and lower zone involvement

The presence at the CXR of all three predictors was associated to a positive CXR in 95.3% of cases, the presence of two of them to 89.2%, of one to 40.7%, and their absence to 7.4%. The presence of at least two of the above-mentioned characteristics resulted in CXR+ in 92.5% of cases. Figure 3 illustrates the 95%CI for the Odds Ratios for positive CXR in presence of all 3 prognostic favors, of 2, of 1 and of none. In patients with both LDH > 500 and CRP > 30, in 74% of cases, at least 4 days had elapsed since the onset of symptoms. Figure 4 illustrates two cases without (A and B) and two with (C and D) predictor factors (Table 6).

**Table 6:** Comparison among studies on CXR sensitivity in COVID19 patients

	No. of patients	CXR sensitivity (%)	95% confidence interval (%)
Wong et al.	64	68.8	55.9–79.8
Guan et al.	274	59.1	53.1–65.0
Weinstock et al.	636	41.7	37.8–45.6
Cozzi et al.	234	68.1	61.6–73.9
Ippolito et al.	204	57.0	47.0–67.0
Schiaffino et al.	408	89.0	85.5–90.9
Our study	260	61.2	54.9–67.1

## Discussion

Our study investigated the role of CXR in patients with COVID-19 and its association with clinical and laboratory data. The main findings may be summarized as follow:

- CXR when compared with RT-PCR has a sensitivity of 61% (95%CI 55–67%) and, when positive, it usually shows the presence of bilateral airspace opacities with peripheral distribution and predominant involvement of the lower lobes.
- Several clinical and laboratory data are associated with the outcome of CXR. The most significant ones LDH and CRP time interval between the onset of symptoms and the CXR.

To the best of our knowledge, there are only seven papers in the literature, excluding case reports and case series, evaluating the sensitivity of CXR in COVID-19 patients. Table 4 compares our results on sensitivity of CXR with those of the authors who performed similar estimates [7–12]. We found a sensitivity of CXR of 61.1% (95%CI 55–67%) in the identification of abnormalities in COVID-19 patients. Five studies, including ours, yield consistent 95%Confidence Intervals [7, 9–11]. There are two exceptions, at the two ends of the spectrum of values: the lower value reported by Weinstock et al. [8], relative to patients admitted to urgent care centers, therefore likely to have a lower grade pathology, and the higher value reported by Schiaffano et al. [12] for patients hospitalized in the Lombardy region, which might be due to the higher-grade pathology present in that region. Overall, the CXR is characterized by relatively low sensitivity in the identification of pulmonary alterations of COVID-19.

As for radiological findings, according to recent literature data, our study described a scenario superimposable on the one described for CT with the presence of peripherally distributed, bilateral opacity with prevalence in the lower lobes [14, 15] and low incidence of pleural effusion.

Regarding relationship with clinical symptoms both univariate and multivariate analysis of our sample of patients underlined a significant difference between CXR+ and CXR- in the time elapsed between the onset of symptoms and the execution of CXR; in particular patients who had a negative result performed CXR at a median of 4 days after the onset of symptoms, about 3 days sooner than the patients with positive CXR.

The COVID-19 patients are known to have a dynamic radiological pattern which varies with their clinical evolution. Four stages of lung involvement were defined for CT [14]: (1) early stage (0–4 days after initial symptoms), with ground glass opacity (GGO) representing the main radiological demonstration; (2) progressive stage (5–8 days after the onset of symptoms), with a worsening of pulmonary involvement and presence of diffuse GGO, crazy-paving pattern and consolidation; (3) peak stage (9–13 days after the onset of symptom) with prevailing dense consolidation is prevalent in association with other findings; (4) absorption stage (≥ 14 days after the onset of the initial symptoms) in which the consolidation is gradually absorbed and no crazy-paving pattern is present.

Wong et al. [7] reported that also the findings at CXR changed over time, reaching the peak stage at 10–12 days from the onset of symptoms. This means that our CXR- patients, who had a median interval of 4(1–7) days between initial symptoms and CRX, were in the “early stage” of the disease, characterized by the presence of GGO, which may be extremely difficult to detect on CXR [16]. Our data emphasizes the concept that particularly in the early stages of the disease, CXR has a low sensitivity for COVID-19. However, this clinical-radiological delay may also be useful to address a differential diagnosis with “classical” community-acquired pneumonia, in which the alterations become manifest in the CXR within a time interval of 12 h from the beginning of the symptomatology [17].

Regarding laboratory data, a marked reduction in lymphocytes and elevation of the concentrations of CRP, LDH and hepatic enzyme are often observed in COVID-19 patients. Recently, a few laboratory features were reported to be associated with severe disease in COVID-19 patients [1, 9, 18, 19]. In a study of more than 1000 patients, Guan et al. [9] showed that among the laboratory parameters that assessed inflammation and cell damage, CRP and LDH were significantly higher in patients with a severe disease than in patients with a non-severe disease and thus appeared to have a prognostic impact.

A recent study [19] found that LDH can be recognized as an important predictive factor for severe COVID-19 manifestations. It must be emphasized that during the 2009 influenza A (H1N1) pandemic, 77.8% of patients whose laboratory data indicated elevation of LDH, had lung involvement, suggesting that LDH elevation was associated with multiple pathogenic factors including viruses, and was important to lung injury [20]. In addition, Henry et al. [19] recently demonstrated that elevated LDH values were associated with the risk of developing severe disease (sixfold increase) and mortality (16-fold increase).

CRP level significantly increases in COVID-19 patients due to inflammatory reaction and tissue destruction. High concentrations of CRP were reported to indicate more severe illness-, associated with lung damage and worse prognosis [21, 22]. In addition, CRP values in other viral diseases, such as H1N1 influenza, were higher for patients with a serious history of the disease [23].

According to our results, LDH and CRP are major predictors of a positive CXR: in presence of both values above the respective threshold of 30 mg/L and 500 U/L respectively, the CXR is positive in about 90% of the patients. This is also in line with the findings of Guan et al. [9] on the higher frequency of positive CXR in patients with a more severe disease.

The overall scenario of our findings suggests that baseline CXR, when integrated with laboratory evaluations, can have a role in the identification of patients with more severe involvement of the pathology. This integrated approach may be a valid alternative when or where other more specific tests (*in primis* RT-PCR tests) are limited. To this end, an added value comes from the promising artificial intelligence techniques developed for improving the diagnostic accuracy of imaging and assisting radiologists and clinicians in the CXR evaluation as part of the COVID-19 triage process [24]. The results of our study should also warn that when dealing with the suspicion of a positive COVID-19 in a patient admitted to the emergency room a few days after the onset of symptoms and without severe alterations of CRP and LDH, physicians should not be surprised to be faced with a negative CXR.

This study has some limitations. First, it is a retrospective study on a limited number of patients, even if it is one of largest in the literature dealing with sensitivity of CXR. Second, we only assessed the sensitivity of the CXR without evaluating its specificity and predictive values by comparing it to a non-COVID-19 control group. Third, we did not correlated the outcome of CXR with the clinical outcome and this should be the goal of further prospective studies that effectively assess the additional role of CXR in patients with suspected SARS-CoV-2 infection.

In conclusion, the baseline CXR performed on 260 patients with COVID-19 confirmed by RT-PCR has a sensitivity of 61.1% with a typical presence of bilateral airspace opacification more often with a lower zone and peripheral distribution. Among demographic characteristic, comorbidities, clinical and laboratory data, LDH > 500 U/L and CRP > 30 mg/L and an interval between the onset of symptoms and the execution of CXR of more than 4 days are the major predictors for a positive CXR.
